# Tonsilitis

**Published:** 1910-04

**Authors:** Charles J. Drueck

**Affiliations:** Chicago, Ill., Professor of Physiology at the Illinois School of Dentistry. Lecturer to the Nurses of Mercy Hospital


### Tonsilitis.

BY CHARLES J. DRUECK, M. D., CHICAGO, ILL.,

Professor of Physiology at the Illinois School of Dentistry. Lecturer to the Nurses of Mercy Hospital.

In the treatment of tonsilitis it is well to remember that this disease is at first only a local disturbance and if promptly and efficiently treated will remain so.

The systemic symptoms—fever, headache, etc.—only develop when there is considerable infection taken up. Therefore, the following course should be instituted early in the case. The first indication is to increase local circulation, and the best therapeutic agent is heat. In the first place confine the patient as much as possible to the house. Children should be put in bed. By staying indoors the patient breathes warm air only, usually free from dust and other irritating substances. The external application of the hot water bag greatly increases the venous circulation and so relieves the congestion, as does also the drinking of hot water. This drinking bathes the parts as well as adding a large amount of water to the bowels and so increases the action of both bowels and kidneys, and washes out the infection as it is taken up by the system, the drinking of water also increases arterial tension which prevents stasis.

A local remedy must fill two requirements. A detergent antiseptic, and a degree of permanency of effect. Many of the remedies are antiseptic, but they are not exosmotic enough to increase the circulation, or else their effect is too transient and their use tires the patient. Locally I have grown to use but one remedy and that is Glyco-Thymoline. I prescribe equal parts of Glyco-Thymoline and water to be used in an atomizer. I get better results with this than anything else I have used. I always use it in an atomizer because gargling is necessarily painful, while a spray is not. Glyco-Thymoline promptly relieves the dry congested condition, and by adhering to the tonsil protects it from external irritation. Its anodyne effect is immediate and lasting. I instruct my patient to use it frequently and because it is pleasant and its action prompt, I find that they need no other instruction but use it thoroughly. As Glyco-Thymoline is non-poisonous, it makes no difference as to how much is swallowed and its action does not upset the stomach, but tends rather to assist the destruction of any of the plugs that may be swallowed.

I find by this method of treatment that my cases are nearly all cured in twenty-four to thirty-six hours. That I need no other medicament at all, because the system does not become clogged with toxines.

I report below two cases, not for their individuality, but because their prototypes are constantly occurring to every physician.

Baby J., child 6 years old, had been sick for two days and the previous day the mother had seen sore throat and treated it with salt, vinegar, etc., to which the child rebelled. When seen, I put child on spray of equal parts Glyco-Thymoline and hot water and allowed sipping of hot soups and liquids; advised use of spray every half to one hour. Next morning mother telephoned I need not come as the child was perfectly well.

Mr. H. K., subject to repeated attacks of tonsilitis, but refuses tonsillotomy because he is afraid it may injure his voice—he is a vocalist. Several months ago I recommended spraying the throat with Glyco-Thymoline one-third strength, twice daily and whenever the throat is at all sore to use it frequently. He has not had an attack of sore throat all this winter.

